# Clinical Implications for the Timely Diagnosis of* Mycobacterium marinum* in the Age of Biologic Therapy: A Case Report and Review of the Literature

**DOI:** 10.1155/2017/5274302

**Published:** 2017-03-14

**Authors:** Chris J. Lata, Kelle Edgar, Stephen Vaughan

**Affiliations:** ^1^Department of Medicine, Cumming School of Medicine, University of Calgary, Calgary, AB, Canada; ^2^Division of Infectious Diseases, Department of Internal Medicine, University of Calgary, Calgary, AB, Canada

## Abstract

*Mycobacterium marinum* infections typically present as cutaneous nodular lesions with a sporotrichoid lymphatic spread on extensor surfaces of extremities. The natural history of this infection can be altered if the host is immunosuppressed, leading to disseminated presentations. A detailed exposure history and high degree of suspicion for this indolent pathogen are often required for the correct diagnosis of this disease. We present a case of a 67-year-old male misdiagnosed with seronegative rheumatoid arthritis presenting with rheumatic nodules. Initiation of chronic immunosuppressant therapy including biologic monoclonal antibodies resulted in the exacerbation of initially localized disease to broadly disseminated lymphatic, joint, and myotendinous granulomatous disease and led to delay in the correct diagnosis. Cessation of immunosuppressants, with a prolonged course of antimicrobial therapy and multiple surgical debridements were required for cure.

## 1. Introduction

*Mycobacterium marinum* is a fastidious nontuberculous mycobacteria (NTM) that causes indolent granulomatous infection which is often difficult to diagnose [1]. This slow-growing species resides in both fresh and salt water environments with optimal growth at 30–32°C [2]. It is carried by many fish species and can result in human infection via inoculation of the skin by fish bite or exposure of an open wound to contaminated water [3].* M. marinum* infections commonly present as soft tissue infections of the extremities, appearing as first as solitary red-violet nodules with a sporotrichoid spreading pattern. Granulomatous nodules can ulcerate or spread infection to underlying bones and joints [4, 5], which can present as arthritis or synovitis, often of the hand or wrist [6].

*M. marinum* is commonly responsive to monotherapy with clarithromycin, trimethoprim-sulfamethoxazole, minocycline, and doxycycline. However, it is most often treated with combination antibiotics such as ethambutol and rifampin, concomitant with source control via surgical debridement [7].

## 2. Case Presentation

A previously healthy 67-year-old male presented with a swollen left hand following a laceration to his third digit after working in soil in his yard in June 2013. He also managed three large hobby aquariums. A 10-day consecutive course of cefprozil and clindamycin marginally improved his swelling. However, he returned in October 2013 with worsening stiffness and digital and hand swelling, which resulted in a diagnosis of seronegative rheumatoid arthritis (RA) after consultation with rheumatology. All autoimmune serology was negative, and inflammatory markers were normal. His swollen left hand partially resolved after pulsed dose prednisone (20 mg daily/2 weeks) but worsened again. He then received prednisone (10 mg/day) and sulfasalazine (2 g/day) with incomplete improvement followed by progression with spread to his left elbow. Several aspirations and intra-articular steroid injections of left elbow, wrist, and MCP joints were attempted with minimal improvement. On the presumption that his rheumatoid arthritis was refractory, methotrexate and leflunomide were added.

In January 2014, the patient evolved nodules located on the extensor surfaces of his left arm and hand. New nodules appeared on the ulnar aspect of his thumb, radial aspect of his index finger, styloid process, and mid shaft of the ulna. Due to ongoing disease, the biologic adalimumab (Humira) was started in February 2014. Prednisone and sulfasalazine were discontinued, but methotrexate and leflunomide were continued. Infectious prescreening for his biologic therapy screening occurred one week after receiving the first dose of Humira. A Mantoux test read at 9-millimetres was presumed to be falsely positive after a subsequent negative interferon-gamma release assay (IGRA) test (Quantiferon Gold™ In-Tube) was obtained.

After three months of treatment, he continued to have a swollen left olecranon and developed lower extremity nodules also. Several joint aspirations were performed from his wrist and knee (due to swelling), but microscopy and bacterial cultures were negative.

**Figure 1:**
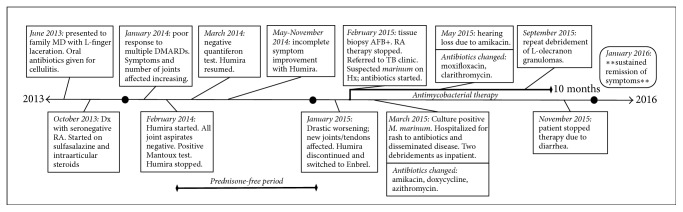
Summary timeline of patient clinical course and treatments.

In December 2014 he had worsening of the swelling and pain in the left wrist, metacarpophalangeal joints, elbow, and left knee. Sulfasalazine and prednisone were restarted along with rotation of his biologic to etanercept. This resulted in dramatic worsening of his nodular lesions. An urgent biopsy of a left arm nodule and a synovial fluid aspirate of his left elbow were collected and were both positive for acid-fast bacilli consistent with mycobacterium. His immunosuppressants were immediately discontinued and he was urgently referred to the Tuberculosis clinic. Subsequently, the mycobacterial species was determined by mycobacterial growth indicator tube (MGIT) culture to be* Mycobacterium marinum* in two additional joint aspirates (left wrist and olecranon bursa) that were grown at reduced temperature. A summary of the patient's clinical course is depicted in Figure 1.

## 3. Treatment Course

Initial treatment included oral azithromycin 250 mg once daily (OD), rifampin 600 mg OD, ethambutol 1000 mg OD, and levofloxacin 750 mg OD. Four weeks later the patient's lesions had worsened, with blistering and weeping developing on his olecranon bursa and 4th digit wounds. Levofloxacin was exchanged for sulfamethoxazole-trimethoprim 2 double-strength tablets twice daily (BID), but he returned two weeks later with a generalized pruritic maculopapular rash secondary to drug reaction. All medications were then discontinued. However, the patient's left upper extremity had evolved to be extremely swollen with serous discharge, prompting admission for ongoing management.

**Figure 2:**
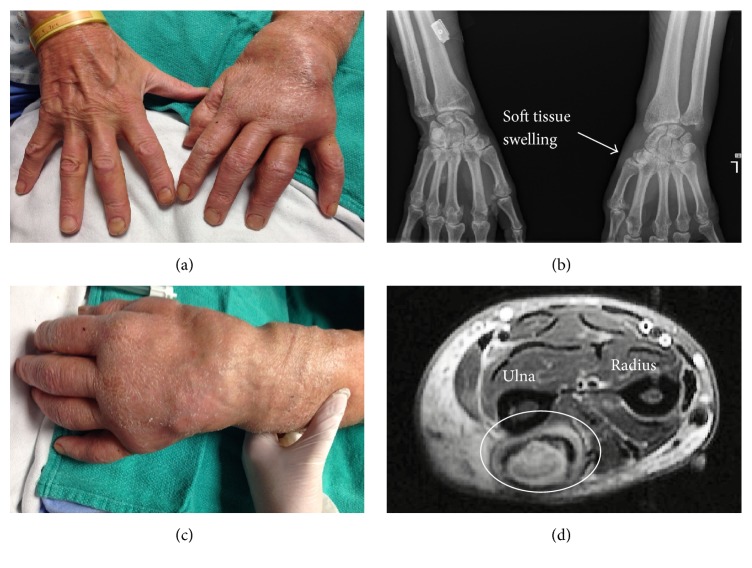
Sporotrichoid spreading of* M. marinum* via lymphatic and myotendinous spreading in disseminated disease. (a) Unilateral granulomatous disease with soft tissue swelling due to lymphatic obstruction, (b) radiograph demonstrating unilateral left soft tissue swelling in absence of erosive disease, (c) dorsal aspect of distal left forearm with visible nodules, and (d) MRI demonstrating myotendinous necrotic granuloma (white circle) in distal aspect of extensor carpi ulnaris.

**Figure 3:**
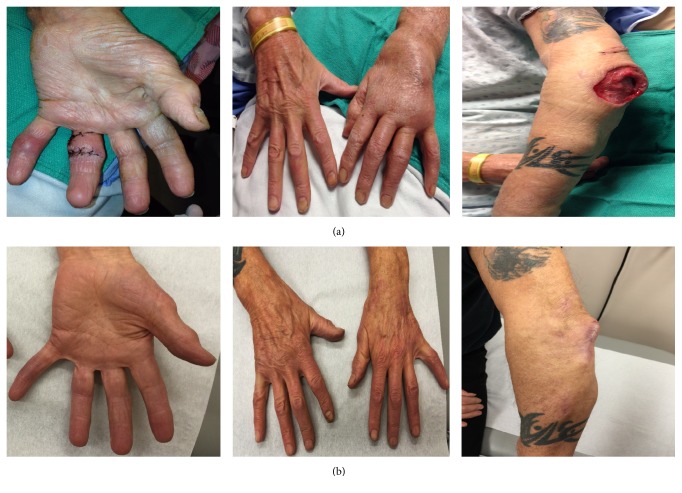
Pre- and postantibiotic therapy photos of lymphatic granulomatous disease from disseminated* M. marinum* infection. (a) preantimicrobial therapy and (b) posttherapy cure (left-to-right: palmar inflamed granulomas 4th/5th digits and resolution, dorsal lymphogranulomatous spread with soft tissue swelling of the left hand and corresponding resolution photo, surgical excision of left elbow granulomatous bursitis, and resolved inflammation with remnant scarring).

On admission, he was systemically stable with a mild leukocytosis (13.1 × 10*E*9/L). Oral doxycycline 100 mg BID and azithromycin 250 mg OD with intravenous Amikacin 1000 mg OD were initiated with rapid improvement of nodular disease (Figure 2). Four surgical debridements of his left upper extremity were required, along with a change of his antibiotics to clarithromycin 500 mg BID and moxifloxacin 400 mg OD due to amikacin-related hearing impairment. He completed 10 months of therapy with full resolution of his nodular disease. No symptoms of inflammatory arthritis recurred in this time (no further immunosuppressive therapy was given) (see Figure 3).

## 4. Discussion

Infection due to* M. marinum* is often overlooked due to its indolent nature. A review of the literature discovered other cases that were mistakenly treated as RA [8, 9] or were presumed to be refractory disease in patients on immune suppressant medications for inflammatory disorders [10–12]. Although persistent infection with NTM is relatively rare in immunocompetent hosts, the increasing use of immunosuppressive therapy has seen increased incidence of these diseases [13]. Several aspects of our patient's presentation revealed subtle clues to an infectious etiology. His presumed RA was seronegative, suggesting the possibility of alternative diagnoses, as rheumatoid nodules are rare (only 6% of cases) in seronegative presentations [14]. Also, there were multiple opportunities for mycobacterial culture from olecranon/nodule samples, especially given the severely refractory nature of this presentation as his immune suppression led to a dissemination of infection.

Our patient was already taking steroids and had received a dose of biologic immunosuppressive medication prior to his TB skin test. This test was positive but was misinterpreted based on a history of prior BCG vaccination. Follow-up IGRA, which has lower cross-reactivity for NTM organisms, was negative. The Mantoux test is sensitive for latent infection by* Mycobacterium* species in the* Mycobacterium tuberculosis* complex, although the test has known cross-reactivity for NTM (including* M. marinum*) [15, 16]. Further, initial partial resolution of symptoms with biologics was likely misinterpreted as successful treatment but was likely due to prednisone discontinuation (see Figure 1).

In summary,* M. marinum* infection classically presents as a nodular granulomatous skin infection of the extremities with sporotrichoid pattern. A localized infection can disseminate via lymphatics in states of immune suppression. Given that rheumatoid nodules are uncommon in seronegative RA, NTM infections need to be considered prior to diagnosis and the implementation of chronic immunosuppressive therapies, particularly biological agents. A thorough environmental exposure history should be conducted with consideration of infectious etiologies in chronic skin conditions. Mantoux and IGRA testing should be conducted before initiation of immunosuppressant medication, although negative results do not reliably rule out known cross-reacting species such as* M. marinum* and present the risk of being interpreted as falsely negative. Positive results, particularly in patients with no known risk factors for latent tuberculous infection, should prompt consideration of NTM infection in the differential.
